# Relationship between vertical and horizontal force-velocity-power profiles in various sports and levels of practice

**DOI:** 10.7717/peerj.5937

**Published:** 2018-11-13

**Authors:** Pedro Jiménez-Reyes, Pierre Samozino, Amador García-Ramos, Víctor Cuadrado-Peñafiel, Matt Brughelli, Jean-Benoît Morin

**Affiliations:** 1 Faculty of Sport, Catholic University of San Antonio, Murcia, Spain; 2 Centre for Sport Studies, King Juan Carlos University, Madrid, Spain; 3 Laboratoire Interuniversitaire de Biologie de la motricité (EA7424), University of Savoie Mont Blanc, Le Bourget du Lac, France; 4 Department of Physical Education and Sport, Faculty of Sport Sciences, University of Granada, Granada, Spain; 5 Department of Sports Sciences and Physical Conditioning, Faculty of Education, CIEDE, Catholic University of the Most Holy Concepción, Concepción, Chile; 6 Department of Health and Human Performance, Polytechnic University of Madrid, Madrid, Spain; 7 Sports Performance Research Institute New Zealand, Auckland University of Technology, Auckland, New Zealand; 8 Laboratoire Motricité Humaine Education Sport Santé, Université Côte d’Azur, Nice, France

**Keywords:** Jumping, Sprinting, Testing, Force–velocity profile, Strength training

## Abstract

This study aimed (i) to explore the relationship between vertical (jumping) and horizontal (sprinting) force–velocity–power (FVP) mechanical profiles in a large range of sports and levels of practice, and (ii) to provide a large database to serve as a reference of the FVP profile for all sports and levels tested. A total of 553 participants (333 men, 220 women) from 14 sport disciplines and all levels of practice participated in this study. Participants performed squat jumps (SJ) against multiple external loads (vertical) and linear 30–40 m sprints (horizontal). The vertical and horizontal FVP profile (i.e., theoretical maximal values of force (*F*_0_), velocity (*v*_0_), and power (*P*_max_)) as well as main performance variables (unloaded SJ height in jumping and 20-m sprint time) were measured. Correlations coefficient between the same mechanical variables obtained from the vertical and horizontal modalities ranged from −0.12 to 0.58 for *F*_0_, −0.31 to 0.71 for *v*_0_, −0.10 to 0.67 for *P*_max_, and −0.92 to −0.23 for the performance variables (i.e, SJ height and sprint time). Overall, results showed a decrease in the magnitude of the correlations for higher-level athletes. The low correlations generally observed between jumping and sprinting mechanical outputs suggest that both tasks provide distinctive information regarding the FVP profile of lower-body muscles. Therefore, we recommend the assessment of the FVP profile both in jumping and sprinting to gain a deeper insight into the maximal mechanical capacities of lower-body muscles, especially at high and elite levels.

## Introduction

Maximal power output is a widely accepted muscular determinant of jumping and sprinting performance, which is determined by both force and velocity production capabilities (Samozino et al., 2012, 2016). Therefore, the performance in jumping (i.e., jump height) and sprinting (i.e., time to run a given distance) mainly depends on the ability of athletes’ neuromuscular and osteo-articular systems to (i) generate high levels of force, (ii) ensure the effective application of this force onto the environment (i.e., supporting ground) and (iii) produce this effective force at high contraction velocities (Morin & Samozino, 2016).

Previous studies have investigated the extent to which jumping tests could predict sprint performance and how sprint and jump performances were correlated (Cronin, Ogden & Lawton, 2007; Randell et al., 2010; Comfort et al., 2013). These studies generally revealed significant correlations between the performance in traditional jump tests (e.g., squat jump (SJ) and countermovement jump) and sprinting performance. An increase in overall lower limb strength after different types of resistance training has also proven to be effective to enhance sprint performance in different populations, ranging from physically active but not trained subjects to professional athletes (Blazevich & Jenkins, 2002; Loturco et al., 2015; Ramírez-Campillo et al., 2015). These results are supported by the meta-analysis of Seitz et al. (2014) that showed a very large significant relationship (*r* = −0.77) between the changes in squat one repetition maximum (1RM) and sprint times. However, it should be noted that squat 1RM, jump height achieved against a single load, or sprint times did not provide comprehensive information about the muscular determinants of performance compared to the entire force–velocity–power (FVP) continuum/spectrum (Morin & Samozino, 2016).

The assessment of the linear force–velocity relationship has been used to identify the maximal mechanical capabilities of the muscles involved to generate high level of force (through the theoretical maximal force, *F*_0_), to generate force at very high velocity (through the theoretical maximal velocity, *v*_0_), and to produce maximal power (*P*_max_). The vertical jump has been the task most commonly used to assess the FVP profile of lower-body muscles (Jiménez-Reyes et al., 2014; Samozino et al., 2014; García-Ramos et al., 2017). The main reasons for the popularity of vertical jumps is their ease of use, high reliability of mechanical outputs, and similarity between jumping movement patterns and some athletic skills (Yamauchi & Ishii, 2007; Nagahara et al., 2014). Recently, a methodology has also been proposed to determine the FVP profile in sprinting (Samozino et al., 2016; Cross et al., 2017). Therefore, nowadays, strength and conditioning practitioners may use field methods for assessing the FVP profile in both jumping (Samozino et al., 2008; Giroux et al., 2014; Jiménez-Reyes et al., 2017b) and sprinting (Samozino et al., 2016; Romero-Franco et al., 2017). These innovative approaches of FVP profiling provide researchers and sport practitioners a simple yet accurate solution for more individualized monitoring and training of physical and technical capabilities (Morin & Samozino, 2016). It is important to note that the FVP profile in jumping (vertical profile) gives information on lower limb capability to produce force in a vertical direction, while the FVP profile in sprinting (horizontal profile) gives information about the capability to produce force which is effective for a specific sprinting task (i.e., horizontal force). However, the degree to which the same maximal mechanical outputs (i.e., *F*_0_, *v*_0_, and *P*_max_) are related between both tasks remains unknown.

The field methods proposed by Samozino et al. (2012, 2014) to assess the FVP profile have recently been used to better understand and improve vertical jumping (Jiménez-Reyes et al., 2017a) and sprint performance (Cross et al., 2015; Rabita et al., 2015; Mendiguchia et al., 2016; Samozino et al., 2016; Slawinski et al., 2017). These studies provide an emerging database of reference values for the mechanical components of the vertical and horizontal FVP profiles. However, these reference data include only some sports (soccer, rugby, athletics) in either the vertical or the horizontal profile, and only for a limited number of levels of practice (e.g., horizontal FVP profile characteristics of elite sprinters in Slawinski et al. (2017). While an optimal FV profile exists in jumping and can be used as an individual reference to monitor training regimen for acyclic ballistic movement performances involving athlete’s own body mass (Jiménez-Reyes et al., 2017a), no individual optimal values exist for other kinds of ballistic movements (e.g., performed against resistances) and for sprint FVP profile yet. Thus, two main research and practice questions remain unanswered regarding the use of the FVP profiling: are vertical and horizontal FVP profiles related, depending on the sport and level of practice? What values may be considered as references when assessing/monitoring athletes’ FVP profiles? The first question has only been addressed with elite female soccer players showing very large correlations for *P*_max_ (*r* = 0.75) but trivial-moderate correlations for *F*_0_ (*r* = −0.14) and *v*_0_ (*r* = 0.49) (Marcote-Pequeno et al., 2018), while the second question has only received partial answers (i.e., few levels of practice in a limited number of sports). It should be noted that the data presented by Marcote-Pequeno et al. (2018) were also considered for the database of the current study.

To address the existing gaps in the literature, the first aim of this study was to test the relationship between vertical (jumping) and horizontal (sprinting) FVP mechanical outputs (*F*_0_, *v*_0_, and *P*_max_) and performance variables (SJ height and sprinting time to 20 m) in a large number of sports and for various levels of practice (leisure to elite). A secondary aim was to provide a large database (>500 individuals) to serve as reference values for these FVP profile characteristics in all the sports and levels tested. Such information would be of interest for both researchers and sport practitioners in order to compare their subjects/athletes FVP profiles to the reference values as well as to elucidate whether the association between vertical and horizontal FVP profiles could be affected by the sport modality and the level of practice.

## Method

### Participants

A total of 553 athletes (333 men (age = 23.5 ± 5.2 years (range = 16–34 years); body mass = 77 ± 13 kg; stature = 1.80 ± 0.08 m) and 220 women (age = 23.2 ± 4.5 years (range = 16–33 years); body mass = 60 ± 8 kg; stature = 1.67 ± 0.07 m)) from various sport disciplines and levels of practice volunteered and gave their written informed consent to participate in this study, which was approved by the local ethical committee of the Catholic University of San Antonio (Murcia) in agreement with the Declaration of Helsinki. The volunteers were split into the following sub-groups: Low Level or amateur (16 men and 14 women; no competing at any level (e.g., sport sciences students, 3–5 h of weekly training volume), medium level or semi-professional (96 men and 105 women; subjects following a structured training (about 10–12 h of weekly training volume) competing at third division leagues or similar categories in their sports), high level or professional (95 men and 45 women; subjects who are professionals in their sports and competing at high level in first and second division leagues or similar categories in their sports (about 18–20 h of weekly training volume)), and elite level or international (126 men and 56 women; subjects who are professionals and also competing at international level with their teams or national teams). The study protocol was approved by the Institutional Review Board of the Catholic University of San Antonio (no: 171114).

### Experimental design

The present work consisted of a cross-sectional design where individual FVP profiles (in both jumping and sprinting modalities) of male and female participants from different sport disciplines and levels of practice were assessed. For all participants, jump and sprint tests were performed over two different testing sessions, within the same week, to avoid the effects of fatigue and ensure the same state of physical fitness. In addition, the tests were conducted at the same time of day in the two testing sessions.

### Testing procedures

#### Force–velocity–power profile in jumping

All sessions began with a general warm-up consisting of jogging and joint mobility exercises, which was followed by a specific warm-up comprising several trials of both unloaded and loaded SJs. Once the warm-up was completed, participants performed maximal SJ without external loads and against five to eight external loads ranging from 10 to 90 kg to determine the individual FVP relationships (Jiménez-Reyes et al., 2017a). The loads were applied in a randomized order. Before each SJ, participants were instructed to stand up straight and still on the center of the jumping area. Thereafter, they squatted until they reached approximately a 90° knee angle (range: 80–100° knee angle). The initial position was maintained for about 2 s before the initiation of the concentric phase and was controlled by a manual goniometer or an elastic cord to ensure consistent push-off distances across loads. Participants were always instructed to jump as high as possible. Participants kept their hands on the hips for jumps without external loads and on the bar for loaded jumps. Any countermovement was verbally forbidden and carefully checked. If these requirements were not met, the trial was repeated. Two valid trials were performed with each load with 2 min of recovery between trials and 4–5 min between different loads.

The mean values of force, velocity, and power were calculated through the Samozino’s method using three equations based on these simple input variables: system mass (body mass plus external loads), jump height, and push-off distance (Samozino et al., 2008). The push-off distance corresponds to the distance covered by the center of mass during push-off, that is, the extension range of the lower limbs from the starting position to take-off (Samozino et al., 2008). The push-off distance was calculated beforehand for each participant as the difference between the extended lower limb length (iliac crest to toes with plantar flexed ankle) and the height in the individual standardized starting position (iliac crest to ground vertical distance). Jump height was obtained using an optical measurement system (OptoJump Next Microgate, Bolzano, Italy), however, the jump height of sprinters and rugby players was obtained from the flight time with a force plate (OR6-5-2000; Advanced Mechanical Technology, Inc., Watertown, MA, USA). Note that the OptoJump device has shown high validity for measuring jump height compared to force place recordings (Glatthorn et al., 2011; Castagna et al., 2013).

**Figure 1:**
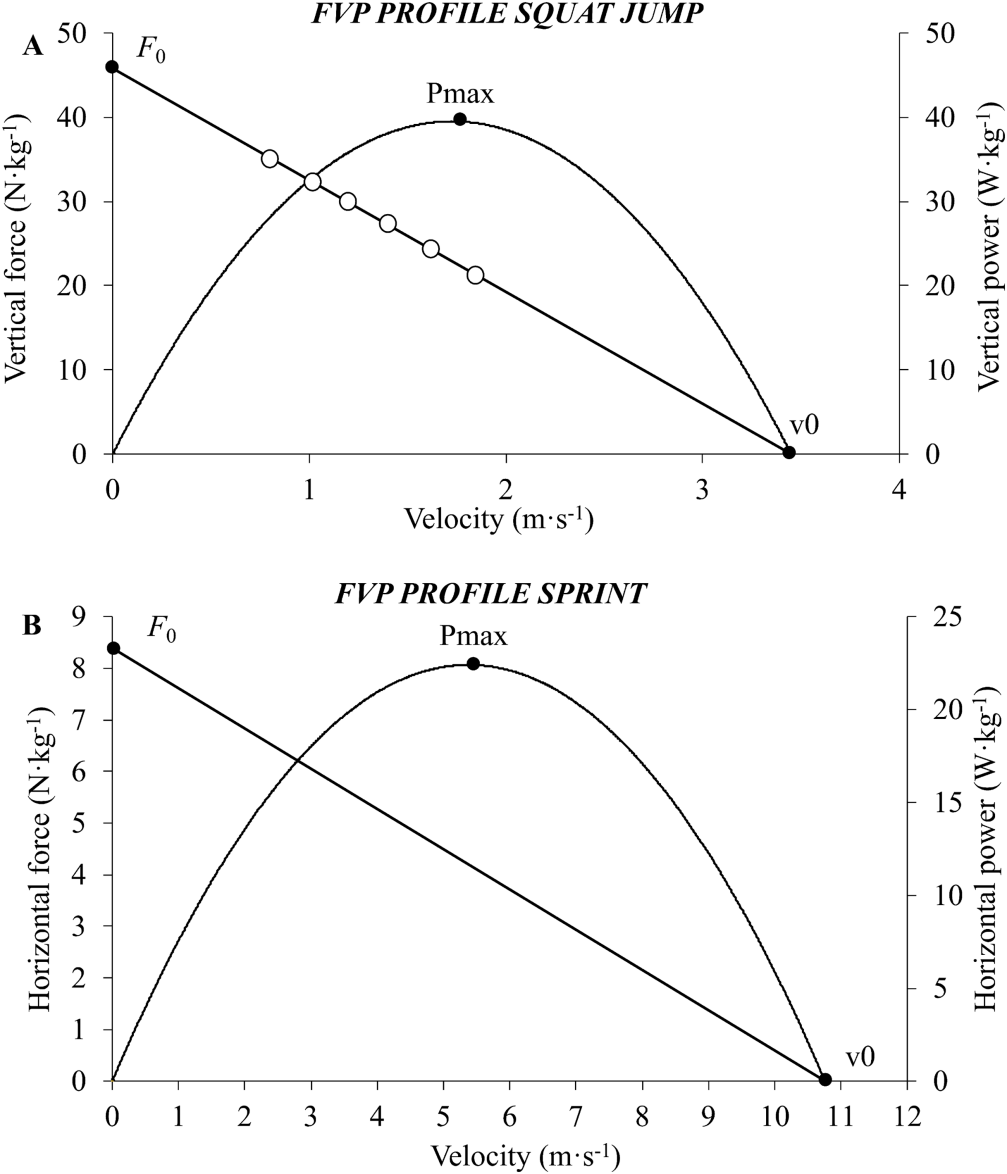
A graphical representation of the force–velocity–power profiles obtained during the jumping (A) and sprinting (B) testing procedures. *F*_0_, theoretical maximal force; *v*_0_, theoretical maximal velocity; *P*_max_, theoretical maximal power.

The values of force and velocity obtained during the trial with higher jump height of each loading condition was modelled by least squares linear regressions to determine the individual FVP profile (Jaric, 2015): *F*(*V*) = *F*_0_ − *aV*, where *F*_0_ represents the maximum force (i.e., force-intercept), *v*_0_ is the maximum velocity (i.e., velocity-intercept), and *a* is the relationship slope of the linear force–velocity relationship (*F*_0_/*v*_0_). As a consequence of the force–velocity relationship being highly linear, the maximum power (*P*_max_) was calculated as *P*_max_ = *F*_0_·*v*_0_/4 (Fig. 1A). *F*_0_ and *P*_max_ were normalized to body mass.

#### Force–velocity–power profile in sprinting

All sessions began with a general warm-up consisting of jogging and joint mobility exercises, followed by three progressive sprints of 30–40 m at increasing running velocities. Subsequently, two to three maximal sprints were performed and the fastest sprint was used for statistical analyses. Running speed during linear sprints of 30–40 m was measured via a Stalker Acceleration Testing System (ATS) II radar device (Model: Stalker ATS II; Applied Concepts, Dallas, TX, USA). The radar device was attached to a tripod 10 m from the starting line at a height of one m corresponding approximately to the height of participants’ center of mass. The radar device sampled velocity–time data at 46.9 Hz, and was operated remotely via connection to a laptop to negate the possibility of variability introduced through direct manual operation. Participants initiated the sprint from a crouched position (staggered-stance).

All data were collected using STATS software (Model: Stalker ATS II Version 5.0.2.1; Applied Concepts, Dallas, TX, USA) provided by the radar device manufacturer. Individual force–velocity relationships in sprinting were assessed as described in previous studies by the Samozino’s method and the theoretical maximum values of force (*F*_0_), velocity (*v*_0_), and power (*P*_max_) were determined (Cross et al., 2015; Mendiguchia et al., 2016; Samozino et al., 2016) (Fig. 1B). Samozino’s method provides a simple method of obtaining the force–velocity relationship from the application of basic laws of motion using the running speed and the body mass of the athlete as main inputs (for full details and validation of the method; please see Samozino et al. (2016). *F*_0_ and *P*_max_ were normalized to body mass. Finally, 20 m time was determined from the modelled velocity–time data.

### Statistical analysis

Descriptive data are presented as means and standard deviations. Pearson’s correlation coefficients (*r*) were used to test the relationship between vertical (*F*_0_-vertical, *v*_0_-vertical, and *P*_max_-vertical) and horizontal (*F*_0_-horizontal, *v*_0_-horizontal, and *P*_max_-horizontal) key variables of the FVP mechanical profile, and performance variables (SJ height and 20 m time). Data were pooled by sport, sex, and level of practice. Qualitative interpretations of the *r* coefficients were provided: trivial (*r* < 0.1), small (*r* = 0.1–0.3), moderate (*r* = 0.3–0.5), large (*r* = 0.5–0.7), very large (*r* = 0.7–0.9), and nearly perfect (*r* > 0.9) (Hopkins et al., 2009). All statistical analyses were performed using SPSS software version 22.0 (SPSS Inc., Chicago, IL, USA) and statistical significance was set at an alpha level of 0.05.

## Results

**Figure 2:**
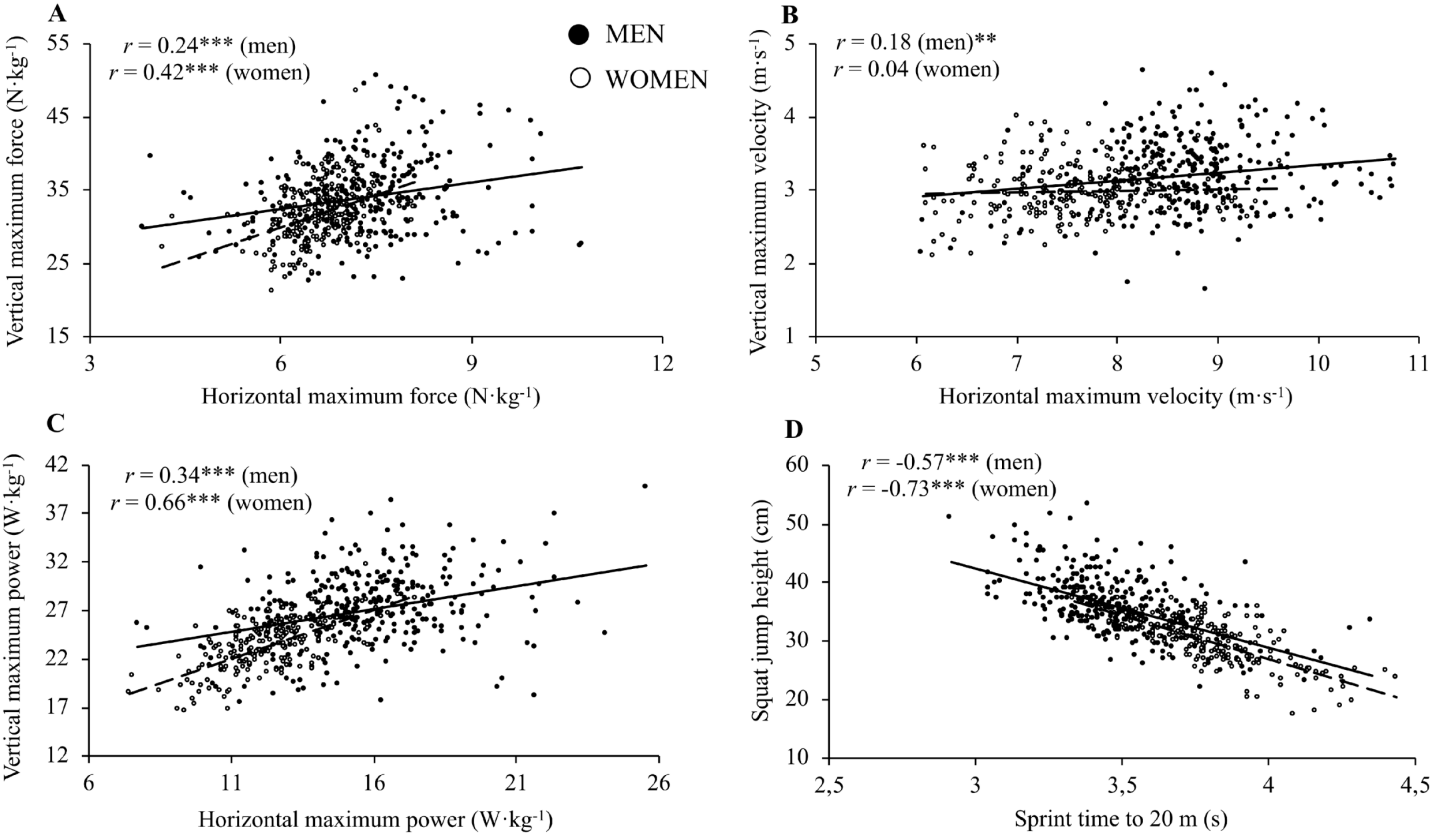
Association between the same force–velocity relationship parameters and performance variables obtained from the jumping and sprinting testing procedures in men (straight line; filled dot) and women (dashed line; empty dot) ((A) force; (B) velocity; (C) maximum power; (D) performance parameters) *r*, Pearson’s correlation coefficient. Significant correlations: **P < 0.01, ***P < 0.001.

The magnitude of the correlations between jumping and sprinting when all data was pooled (*n* = 553) was 0.32 for *F*_0_ (*P* < 0.001) (*r* = 0.24 for men and 0.42 for women; both *P* < 0.001), 0.25 for *v*_0_ (*P* < 0.001) (*r* = 0.18 for men (*P* = 0.001) and 0.04 for women (*P* = 0.572)), 0.55 for *P*_max_ (*P* < 0.001) (*r* = 0.34 for men and 0.66 for women; both *P* < 0.001), and −0.73 for the performance variables (*P* < 0.001) (*r* = −0.57 for men and −0.73 for women; both *P* < 0.001) (Fig. 2). The magnitude of the correlations for specific groups ranged between −0.12 (men elite level handball players) and 0.58 (men sports science students) for *F*_0_, −0.31 (men medium level volleyball players) and 0.71 (women elite level judokas) for *v*_0_, −0.10 (men medium level volleyball players) and 0.67 (women medium level basketball players) for *P*_max_, and −0.92 (women medium level basketball and soccer players) and −0.23 (men elite level futsal players) for the performance variables (Table 1).

**Table 1:** Pearson’s correlations coefficients between the force–velocity relationship parameters (*F*_0_, *v*_0_, and *P*_max_) and performance variables (unloaded squat jump height and 20 m sprint time) obtained during the jumping and sprinting testing.

Sport	*F*_0_ (N/kg)	*v*0 (m/s)	*P*_max_ (W/kg)	Performance
Basketball				
♂HL (*n* = 16)	0.16	−0.07	0.65**	−0.60*
♂ML (*n* = 12)	0.20	0.48	0.25	−0.67*
♀ML (*n* = 17)	0.53*	−0.28	0.67**	−0.92**
All (*n* = 45)	0.33*	−0.26	0.70***	−0.82***
Futsal				
♂EL (*n* = 39)	0.14	0.03	0.02	−0.23
♂HL (*n* = 10)	0.43	0.54	0.49	−0.36
♀ML (*n* = 28)	0.10	0.26	0.34	−0.47*
All (*n* = 77)	0.40***	0.35**	0.70***	−0.74***
Gymnastics				
♂EL (*n* = 14)	0.33	0.35	0.60*	−0.63*
♀EL (*n* = 12)	0.13	0.18	0.45	−0.60*
All (*n* = 26)	0.34	0.56**	0.82***	−0.81***
Handball				
♂EL (*n* = 31)	−0.12	0.31	0.40*	−0.63**
♀EL (*n* = 15)	0.47	0.27	0.47	−0.35
All (*n* = 46)	0.38**	0.01	0.50***	−0.73***
Judo				
♂HL (*n* = 10)	0.60	0.22	0.39	−0.63
♀HL (*n* = 10)	0.46	0.71*	0.59	−0.77**
All (*n* = 20)	0.45*	0.88***	0.65**	−0.87***
Karate				
♂ML (*n* = 18)	0.26	0.42	0.41	−0.46
♀ML (*n* = 14)	0.46	0.29	0.24	−0.67**
All (*n* = 32)	0.24	0.48**	0.61***	−0.81***
Rugby				
♂EL (*n* = 21)	0.01	0.26	−0.01	−0.27
♀EL (*n* = 15)	0.01	0.21	0.09	−0.47
All (*n* = 36)	−0.10	0.28	−0.02	−0.79***
Soccer				
♂EL (*n* = 21)	0.31	−0.05	0.15	−0.32
♂HL (*n* = 18)	0.34	0.15	0.24	−0.45
♂ML (*n* = 34)	0.42*	0.27	0.44**	−0.59***
♀EL (*n* = 18)	0.11	0.06	0.49**	−0.60***
♀ML (*n* = 20)	0.37	0.18	0.63**	−0.92***
All (*n* = 111)	0.47***	0.23*	0.62***	−0.74***
Sprint				
♂HL (*n* = 15)	0.50	0.36	0.36	−0.35
♀HL (*n* = 13)	0.55	0.18	0.26	−0.50
All (*n* = 28)	0.60**	0.55**	0.66***	−0.73***
Taekwondo				
♂ML (*n* = 13)	0.39	0.45	0.21	−0.46
♀ML (*n* = 10)	0.31	0.38	0.10	−0.28
All (*n* = 23)	0.34	0.69***	0.36	−0.59**
Tennis				
♂HL (*n* = 17)	0.50*	0.37	0.41	−0.57
♀HL (*n* = 14)	0.54*	0.61*	0.44	−0.34
All (*n* = 31)	0.43*	0.69***	0.62***	−0.69***
Sports science students				
♂LL (*n* = 16)	0.58*	0.43	0.64**	−0.67**
♀LL (*n* = 14)	0.48*	0.24	0.61*	−0.59*
All (*n* = 30)	0.57**	0.48**	0.78***	−0.83***
Volleyball				
♂ML (*n* = 19)	0.47*	−0.31	−0.10	−0.27
♀ML (*n* = 16)	0.48*	−0.04	0.48	−0.53*
All (*n* = 35)	0.47**	−0.04	0.47**	−0.67***
Weightlifting				
♂HL (*n* = 9)	0.29	0.32	0.49	−0.48
♀HL (*n* = 8)	0.36	0.23	0.46	−0.45
All (*n* = 17)	0.67**	0.22	0.71**	−0.70**

**Notes:**

*F*_0_, theoretical maximal force; *v*_0_, theoretical maximal velocity; *P*_max_, theoretical maximal power; LL, low level or amateur; ML, medium level or semi-professional; HL, high level or professional; EL, elite level or international. Significant correlations (highlighted in bold): *P < 0.05, **P < 0.01, ***P < 0.001. Qualitative interpretations of the Pearson’s correlations coefficients: trivial (*r* < 0.1), small (*r* = 0.1–0.3), moderate (*r* = 0.3–0.5), large (*r* = 0.5–0.7), very large (*r* = 0.7–0.9), and nearly perfect (*r* > 0.9) (Hopkins et al., 2009).

The magnitude of the FVP profile and performance variables classified by sport, sex, and level of practice are summarized in Table 2. These data overall range as follows: *F*_0_-vertical with female sport science students showing the lowest averaged value (25.7 N/kg), whilst male high level weightlifters presented the highest averaged value (47.5 N/kg); *F*_0_-horizontal with the lowest averaged value found in male elite judokas (5.99 N/kg), and the highest in male rugby players (8.90 N/kg); *v*_0_-vertical ranged between the values of female high level judokas (2.54 m/s) to those of male elite handball players (3.69 m/s); the *v*_0_-horizontal values ranged from those of female high level judokas (6.76 m/s) to those of male high level sprinters (10.3 m/s); *P*_max_-vertical ranged between values of female high level judokas (19.7 W/kg) and male high level weightlifters (32.6 W/kg), whereas *P*_max_-horizontal ranged between the values of female high level judokas (10.4 W/kg) and male high level sprinters (20.7 W/kg). Finally, regarding the performance variables, SJ height ranged between 22 cm for female sport science students and 44 cm for male elite level rugby players, while the sprint time to 20 m ranged between 3.14 s for male high-level sprinters and 4.11 s for female high-level judokas.

**Table 2:** Descriptive data (mean ± standard deviation) of the Force–Velocity–Power mechanical profile and performance variables obtained in jumping (vertical) and sprinting (horizontal) testing procedures displayed by sport, sex, and level of practice.

Sport	*F*_0_ (N/kg)		*v*0 (m/s)		*P*_max_ (W/kg)		Performance	
	Vertical	Horizontal	Vertical	Horizontal	Vertical	Horizontal	SJ height (cm)	Sprint time (s)
Basketball								
♂HL (*n* = 12)	32.7 ± 3.78	6.98 ± 1.06	3.37 ± 0.47	8.56 ± 0.40	26.8 ± 3.24	14.8 ± 2.05	0.35 ± 0.05	3.54 ± 0.18
♂ML (*n* = 16)	32.4 ± 3.72	6.71 ± 0.72	3.13 ± 0.43	8.53 ± 0.49	25.3 ± 4.15	14.2 ± 1.34	0.33 ± 0.03	3.58 ± 0.10
♀ML (*n* = 17)	29.7 ± 4.32	6.32 ± 0.61	2.94 ± 0.39	7.19 ± 0.70	21.5 ± 2.10	11.4 ± 1.93	0.27 ± 0.03	3.93 ± 0.26
♂♀HL + ML (*n* = 45)	31.3 ± 4.08	6.66 ± 0.85	3.14 ± 0.46	8.03 ± 0.86	24.4 ± 3.87	13.3 ± 2.39	0.32 ± 0.05	3.70 ± 0.27
Futsal								
♂EL (*n* = 39)	34.8± 3.61	7.70 ± 0.51	3.27 ± 0.45	9.01 ± 0.43	28.2 ± 2.33	17.2 ± 1.35	0.36 ± 0.03	3.36 ± 0.09
♂HL (*n* = 10)	34.4± 2.21	7.11 ± 0.22	3.03 ± 0.21	8.84 ± 0.21	26.0 ± 2.51	15.7 ± 0.54	0.34 ± 0.03	3.46 ± 0.04
♀ML (*n* = 28)	31.7± 2.49	6.63 ± 0.46	2.89 ± 0.27	7.64 ± 0.40	22.8 ± 2.01	12.6 ± 1.18	0.29 ± 0.03	3.77 ± 0.13
♂♀ (*n* = 77)	33.6 ± 3.39	7.23 ± 0.68	3.10 ± 0.40	8.49 ± 0.76	25.9 ± 3.31	15.3 ± 2.46	0.33 ± 0.04	3.52 ± 0.22
Gymnastics								
♂EL (*n* = 14)	35.5 ± 2.23	7.16 ± 0.65	3.35 ± 0.31	8.89 ± 0.36	29.7 ± 2.79	15.8 ± 1.25	0.39 ± 0.04	3.45 ± 0.09
♀EL (*n* = 12)	33.5 ± 2.45	6.83 ± 0.30	3.04 ± 0.24	7.48 ± 0.37	25.3 ± 0.84	12.7 ± 0.71	0.33 ± 0.01	3.78 ± 0.09
♂♀EL (*n* = 26)	34.5 ± 2.50	7.01 ± 0.54	3.21 ± 0.32	8.24 ± 0.80	27.7 ± 3.05	14.3 ± 1.87	0.36 ± 0.04	3.60 ± 0.19
Handball								
♂EL (*n* = 31)	31.7 ± 4.09	7.24 ± 0.70	3.69 ± 1.06	8.54 ± 0.50	28.7 ± 6.78	15.4 ± 1.75	0.35 ± 0.04	3.50 ± 0.13
♀EL (*n* = 15)	31.4 ± 3.26	6.67 ± 0.38	3.08 ± 0.38	7.51 ± 0.31	24.0 ± 1.87	12.5 ± 1.00	0.30 ± 0.02	3.79 ± 0.12
♂♀EL (*n* = 46)	31.6 ± 3.80	7.05 ± 0.67	3.49 ± 0.94	8.21 ± 0.66	27.1 ± 6.05	14.4 ± 2.06	0.34 ± 0.04	3.59 ± 0.19
Judo								
♂HL (*n* = 10)	31.1 ± 2.15	5.99 ± 0.41	3.29 ± 0.23	8.13 ± 0.25	25.5 ± 1.76	12.1 ± 0.58	0.32 ± 0.02	3.76 ± 0.05
♀HL (*n* = 10)	31.2 ± 2.41	6.20 ± 1.26	2.54 ± 0.29	6.76 ± 0.39	19.7 ± 1.64	10.4 ± 2.02	0.25 ± 0.02	4.11 ± 0.22
♂♀HL (*n* = 20)	31.1 ± 2.22	6.09 ± 0.92	2.92 ± 0.46	7.45 ± 0.77	22.6 ± 3.38	11.2 ± 1.69	0.28 ± 0.04	3.93 ± 0.24
Karate								
♂ML (*n* = 18)	35.8 ± 3.64	6.88 ± 1.08	3.13 ± 0.31	8.24 ± 0.45	27.9 ± 2.81	14.0 ± 1.99	0.36 ± 0.03	3.63 ± 0.14
♀ML (*n* = 14)	31.1 ± 1.85	6.84 ± 0.52	2.89 ± 0.33	6.90 ± 0.37	22.4 ± 2.43	11.8 ± 1.27	0.27 ± 0.02	3.93 ± 0.15
♂♀ML (*n* = 32)	33.8 ± 3.78	6.86 ± 0.87	3.02 ± 0.34	7.65 ± 0.79	25.5 ± 3.78	13.1 ± 2.04	0.32 ± 0.06	3.76 ± 0.21
Rugby								
♂EL (*n* = 21)	31.0 ± 7.47	8.90 ± 1.04	3.38 ± 1.13	8.62 ± 0.31	24.7 ± 4.97	19.2 ± 2.36	0.44 ± 0.05	3.29 ± 0.12
♀EL (*n* = 15)	32.9 ± 2.22	7.12 ± 0.40	3.06 ± 0.32	7.85 ± 0.28	24.9 ± 1.48	13.9 ± 1.04	0.33 ± 0.02	3.66 ± 0.07
♂♀EL (*n* = 36)	31.8 ± 5.89	8.16 ± 1.21	3.24 ± 0.89	8.30 ± 0.49	24.8 ± 3.88	17.0 ± 3.25	0.39 ± 0.07	3.45 ± 0.21
Soccer								
♂EL (*n* = 21)	36.7 ± 5.68	7.35 ± 0.69	3.22 ± 0.61	9.25 ± 0.61	28.9 ± 3.16	16.9 ± 1.91	0.38 ± 0.04	3.38 ± 0.12
♂HL (*n* = 18)	35.5 ± 3.19	7.07 ± 0.42	2.98 ± 0.37	9.17 ± 0.49	26.3 ± 2.95	16.1 ± 1.09	0.35 ± 0.04	3.42 ± 0.08
♂ML (*n* = 34)	33.1 ± 4.56	6.73 ± 1.04	3.16 ± 0.52	8.89 ± 0.50	25.7 ± 2.48	14.9 ± 1.72	0.33 ± 0.03	3.50 ± 0.12
♀EL (*n* = 18)	32.9 ± 3.56	6.49 ± 0.30	3.03 ± 0.33	8.18 ± 0.47	24.7 ± 0.95	13.2 ± 1.03	0.33 ± 0.02	3.68 ± 0.10
♀ML (*n* = 20)	31.8 ± 1.84	6.45 ± 0.59	2.88 ± 0.19	7.60 ± 0.38	22.8 ± 1.65	12.2 ± 1.28	0.29 ± 0.02	3.78 ± 0.11
♂♀EL (*n* = 111)	33.9 ± 4.41	6.82 ± 0.64	3.07 ± 0.46	8.67 ± 0.78	25.8 ± 3.09	14.7 ± 2.23	0.34 ± 0.04	3.54 ± 0.18
Sprint								
♂HL (*n* = 15)	40.9 ± 3.12	8.10 ± 0.88	3.11 ± 0.24	10.3 ± 0.44	31.7 ± 3.55	20.7 ± 2.19	0.41 ± 0.04	3.14 ± 0.10
♀HL (*n* = 13)	38.3 ± 2.78	7.07 ± 0.36	2.86 ± 0.22	8.98 ± 0.36	27.3 ± 2.15	15.8 ± 0.91	0.35 ± 0.02	3.45 ± 0.07
♂♀HL (*n* = 28)	39.7 ± 3.19	7.62 ± 0.85	2.99 ± 0.26	9.71 ± 0.80	29.7 ± 3.71	18.4 ± 3.05	0.38 ± 0.05	3.28 ± 0.18
Taekwondo								
♂ML (*n* = 13)	35.6 ± 4.15	6.94 ± 1.91	3.06 ± 0.31	7.89 ± 0.60	27.0 ± 2.80	13.5 ± 3.65	0.35 ± 0.04	3.75 ± 0.33
♀ML (*n* = 10)	33.4 ± 2.21	7.04 ± 0.66	2.56 ± 0.35	6.62 ± 0.38	21.2 ± 1.98	11.6 ± 1.30	0.26 ± 0.02	4.00 ± 0.15
♂♀ML (*n* = 23)	33.8 ± 3.78	6.86 ± 0.87	3.02 ± 0.34	7.65 ± 0.79	25.5 ± 3.78	13.1 ± 2.04	0.32 ± 0.06	3.76 ± 0.21
Tennis								
♂HL (*n* = 17)	31.1 ± 2.10	7.24 ± 0.87	3.48 ± 0.27	8.59 ± 0.33	27.0 ± 2.03	15.4 ± 1.56	0.35 ± 0.03	3.49 ± 0.10
♀HL (*n* = 14)	31.7 ± 1.94	6.92 ± 0.32	3.12 ± 0.24	7.77 ± 0.34	24.7 ± 1.28	13.4 ± 0.93	0.32 ± 0.01	3.69 ± 0.10
♂♀HL (*n* = 31)	31.4 ± 2.01	7.10 ± 0.69	3.32 ± 0.31	8.22 ± 0.53	26.0 ± 2.08	14.5 ± 1.65	0.33 ± 0.03	3.58 ± 0.14
Sport science students								
♂LL (*n* = 16)	28.3 ± 3.11	6.55 ± 0.96	3.67 ± 0.18	8.26 ± 0.53	23.5 ± 3.00	13.4 ± 2.02	0.29 ± 0.04	3.67 ± 0.18
♀LL (*n* = 14)	25.7 ± 2.76	6.12 ± 0.30	3.10 ± 0.33	6.91 ± 0.66	25.7 ± 2.76	10.5 ± 1.11	0.22 ± 0.03	4.04 ± 0.19
♂♀LL (*n* = 30)	27.0 ± 3.19	6.35 ± 0.75	3.22 ± 0.31	7.63 ± 0.90	21.8 ± 3.15	12.1 ± 2.19	0.26 ± 0.05	3.84 ± 0.26
Volleyball								
♂ML (*n* = 19)	32.1 ± 3.12	7.18 ± 0.90	3.46 ± 0.32	8.18 ± 0.50	27.6 ± 2.01	14.6 ± 2.05	0.36 ± 0.03	3.58 ± 0.16
♀ML (*n* = 16)	28.8 ± 2.23	6.62 ± 0.58	3.39 ± 0.34	7.10 ± 0.30	24.3 ± 2.07	11.7 ± 1.14	0.31 ± 0.02	3.89 ± 0.11
♂♀ML (*n* = 35)	30.6 ± 3.19	6.92 ± 0.81	3.43 ± 0.33	7.69 ± 0.69	25.5 ± 3.78	13.3 ± 2.22	0.34 ± 0.04	3.72 ± 0.21
Weightlifting								
♂HL (*n* = 9)	47.5 ± 2.58	7.52 ± 0.53	2.74 ± 0.27	7.67 ± 0.53	32.6 ± 3.80	14.4 ± 1.75	0.43 ± 0.04	3.63 ± 0.17
♀HL (*n* = 8)	38.1 ± 0.76	6.90 ± 0.17	2.77 ± 0.12	7.22 ± 0.30	26.4 ± 0.91	12.4 ± 0.70	0.35 ± 0.01	3.82 ± 0.09
♂♀HL (*n* = 17)	43.1 ± 5.21	7.23 ± 0.51	2.75 ± 0.21	7.46 ± 0.49	29.6 ± 4.21	13.5 ± 1.67	0.39 ± 0.05	3.72 ± 0.17

**Note:**

*F*_0_, theoretical maximal force; *v*_0_, theoretical maximal velocity; *P*_max_, theoretical maximal power; LL, low level or amateur; ML, medium level or semi-professional; HL, high level or professional; EL, elite level or international.

## Discussion

The first aim of this study was to investigate the relationship between the vertical and horizontal FVP profiles across gender and level of practice in a large number of sports, and thus bringing new insights into the debate of interchangeability of “vertical” and “horizontal” (i.e., jumping- and sprinting-based) physical components of sports performance when testing and training. A secondary aim was to provide the associated large database, which may constitute a reference set of data on the topic for researchers and practitioners.

When considering low-level populations, large correlations between vertical and horizontal FVP variables suggest that the ability to develop horizontal force during sprinting is partly associated with the ability of lower limbs to develop force (as assessed during vertical jumps), reflecting the lower limb neuromuscular properties. At these lower levels of practice, we could speculate that training total force production capabilities (via vertical FVP profile) within a resistance training program could be effective at improving sprinting performance. However, in high level to elite populations, horizontal force production during sprinting acceleration is likely less determined by the neuromuscular system capability to produce total force onto the ground as assessed through the vertical FVP profile. Differences in sprinting acceleration performance may be more explained by differences in mechanical effectiveness, that is, the ability to effectively apply the force onto the ground (Morin, Edouard & Samozino, 2011; Morin et al., 2012). This, in turn, would suggest that using the vertical and horizontal FVP profile as interchangeable pieces of information lead to lower errors in low than high level populations.

The results of the present study also suggest that caution should be taken when inferring changes in one skill (e.g., sprinting) as a consequence of an improvement in the other (e.g., jumping). This specificity is also supported by the agreement found between magnitude and orientation of the mechanical outputs analyzed and the sport discipline and level at which it is practiced. When analyzing the existing literature in light of the present results, it is interesting to note that precisely in those studies where the relationship between “horizontal” and “vertical” performances was strong, either low-level athletes were tested (Coutts, Murphy & Dascombe, 2004; Cormie, McGuigan & Newton, 2010) or the tested populations were not sub-categorized by gender, level of practice, or sport discipline, which may have influenced data interpretation (Hammett & Hey, 2003; Deane et al., 2005). In this regard, our results generally revealed larger correlations between the variables of the FVP profile as well as for the performance variables (SJ height and sprint time to 20 m) when the data were not sub-categorized. This clearly raises the question of the “transfer” between strength training and sprint performance, especially in trained and highly-trained athletes (Young, 2006). The present results (i.e., the lower the level and homogeneity of athletes’ groups, the higher the correlation between horizontal and vertical profiles) show that study conclusions should be carefully interpreted in light of the level and homogeneity of the populations tested. Results obtained in low-level heterogeneous groups should not be extrapolated to higher level populations.

Overall, these results also strengthen the need to evaluate *both* jumping and sprinting FVP profiles in order to ensure a more specific, accurate and comprehensive characterization of athletes’ physical qualities, toward better designed training programs. The present study expands the conclusions of Marcote-Pequeno et al. (2018), who reported low correlations for some FVP profile variables between jumping and sprinting in elite female soccer players, to other sport populations and male athletes. Therefore, the FVP approach is expected to be useful for both researchers and coaches, since a more complete knowledge of athletes’ characteristics will very likely facilitate the subsequent prescription of an effective training according to the individual needs (Morin & Samozino, 2016; Jiménez-Reyes et al., 2017a).

Although this study addresses some of the main limitations discussed in the recent literature by Rumpf et al. (2016) and Seitz et al. (2014), such as the grouping of samples from different disciplines and the non-differentiation of both gender and level of practice, it remains cross-sectional in essence. Therefore, the results presented here only apply to the time of evaluation and cannot be extrapolated to training-induced effects and the potential transfer between for instance jumping-type training and sprinting performance (Young, 2006; Ramírez-Campillo et al., 2015). Several studies have recently been conducted to analyze the effects of different types of training on *either* the vertical *or* the horizontal FVP profile (De Lacey et al., 2014; Mendiguchia et al., 2014; Morin et al., 2016), and showed that both profiles were sensitive to specific resistance training. For instance, Jiménez-Reyes et al. (2017a) recently provided evidence of how an individualized training aiming at optimizing the FVP profile is effective at improving jump performance. Studies in preparation will feature a within-subject longitudinal testing of the effects of resistance training on both vertical and horizontal FVP profiles in order to help better discuss this “training transference” phenomenon.

Finally, while acknowledging the limitations expressed, the normative database provided in this study shows the main mechanical FVP variables as well as the performance variables of several popular sports according to athletes’ gender and level of practice. This database may be of interest for a more accurate analysis and monitoring of physical capabilities (Buchheit et al., 2014; Cross et al., 2015; Rabita et al., 2015) and injury-related factors such as horizontal *F*_0_ (Mendiguchia et al., 2014, 2016). In this regard, the database provided in this work confirms previous recommendations of Morin & Samozino (2016) about the need for individualized profiling of both vertical and horizontal mechanics, and the risk taken when inferring one from the other. Finally, it should be noted that the majority of FVP profiles assessed in the present study, with the exception of sprinters and rugby players, were obtained from the flight time recorded by an infrared platform (Optojump). Since infrared platforms are known to underestimate jump height as compared to force plate measurements (Glatthorn et al., 2011), coaches should be aware that the magnitude of the FVP variables could slightly differ when obtained from other measurement methods such as force platforms, linear position transducers, or MySprint2 (Giroux et al., 2014). Future studies should elucidate the effect of the device used to measure jump height on the FVP profile.

## Conclusions

The *P*_max_ and the performance variables (i.e., SJ height and sprint time to 20 m) were the variables more correlated between the jumping and sprinting testing procedures. However, the magnitude of the correlations observed for *F*_0_ and *v*_0_ generally ranged between trivial and small. Interestingly, our results also showed a tendency toward a decrement in the magnitude of the correlations with increasing levels of practice (i.e., the relationship between the variables of both tasks decreased from low level to elite participants). These results suggest that the jumping and sprinting testing procedures could provide similar information, particularly regarding *P*_max_, and performance variables, when assessing low level participants. On the other hand, the low correlations generally observed between the mechanical outputs in high level and elite athletes indicate that the jumping and sprinting testing procedures provide distinctive information regarding the FVP profile of lower-body muscles. Therefore, we recommend the assessment of the FVP profile both in jumping and sprinting to gain a deeper insight into the maximal mechanical capacities of lower-body muscles, especially at high and elite levels.

The two main practical applications of the present study are that (1) it provides reference values of the maximal force-, velocity-, and power-producing capabilities as well as of performance variables (unloaded SJ height and sprint time to 20 m) of athletes of different sport modalities, levels of practice and sex in two important tasks, and (2) it highlights that the FVP profile obtained during one acyclic task as jumping should not be used to infer these mechanical properties of the athletes (and in turn directly design testing or training) in a multi-direction cyclic task as sprinting. Therefore, the FVP profile should be determined with exercises as similar as possible to the targeted performance task.

## Supplemental Information

10.7717/peerj.5937/supp-1 Supplemental Information 1 Database of force-velocity profile from horizontal and vertical modalities in various sports modalities, level and sex. *F*_0_, theoretical maximal force; v0, theoretical maximal velocity; *P*_max_, theoretical maximal power; LL, low level or amateur; ML, medium level or semi-professional; HL, high level or professional; EL, elite level or international.

## Additional Information and Declarations

### Data Availability

The following information was supplied regarding data availability: Raw data are provided in the Supplemental Files.
